# Analysis of exome data for 4293 trios suggests GPI-anchor biogenesis defects are a rare cause of developmental disorders

**DOI:** 10.1038/ejhg.2017.32

**Published:** 2017-03-22

**Authors:** Alistair T Pagnamenta, Yoshiko Murakami, John M Taylor, Consuelo Anzilotti, Malcolm F Howard, Venessa Miller, Diana S Johnson, Shereen Tadros, Sahar Mansour, I Karen Temple, Rachel Firth, Elisabeth Rosser, Rachel E Harrison, Bronwen Kerr, Niko Popitsch, The DDD Study, Taroh Kinoshita, Jenny C Taylor, Usha Kini

**Affiliations:** 1 National Institute for Health Research Oxford Biomedical Research Centre, Wellcome Trust Centre for Human Genetics, University of Oxford, Oxford, UK; 2 Department of Immunoregulation, Research Institute for Microbial Diseases, Osaka University, Osaka, Japan; 3 World Premier International Immunology Frontier Research Center, Osaka University, Osaka, Japan; 4 Oxford NHS Regional Molecular Genetics Laboratory, Oxford University Hospitals NHS Trust, Oxford, UK; 5 The Henry Wellcome Building for Molecular Physiology, University of Oxford, Oxford, UK; 6 Department of Clinical Genetics, Oxford University Hospitals NHS Trust, Oxford, UK; 7 Sheffield Children's Hospital, Western Bank, Sheffield, UK; 8 South West Thames Regional Genetics Service, St George's Healthcare NHS Foundation Trust, London, UK; 9 Human Genetics and Genomic Medicine, Faculty of Medicine, University of Southampton and Wessex Clinical Genetics Service, University Hospital NHS Trust, Princess Anne Hospital, Southampton, UK; 10 Department of Clinical Genetics, Great Ormond Street Hospital for Children NHS Trust, London, UK; 11 Department of Clinical Genetics, Nottingham University Hospitals NHS Trust, Nottingham, UK; 12 Manchester Centre for Genomic Medicine, Institute of Human Development, Faculty of Medical and Human Sciences, University of Manchester, Manchester, UK; 13 Wellcome Trust Sanger Institute, Wellcome Genome Campus, Hinxton, Cambridge, UK

## Abstract

Over 150 different proteins attach to the plasma membrane using glycosylphosphatidylinositol (GPI) anchors. Mutations in 18 genes that encode components of GPI-anchor biogenesis result in a phenotypic spectrum that includes learning disability, epilepsy, microcephaly, congenital malformations and mild dysmorphic features. To determine the incidence of GPI-anchor defects, we analysed the exome data from 4293 parent–child trios recruited to the Deciphering Developmental Disorders (DDD) study. All probands recruited had a neurodevelopmental disorder. We searched for variants in 31 genes linked to GPI-anchor biogenesis and detected rare biallelic variants in *PGAP3*, *PIGN*, *PIGT* (*n*=2), *PIGO* and *PIGL*, providing a likely diagnosis for six families. In five families, the variants were in a compound heterozygous configuration while in a consanguineous Afghani kindred, a homozygous c.709G>C; p.(E237Q) variant in *PIGT* was identified within 10–12 Mb of autozygosity. Validation and segregation analysis was performed using Sanger sequencing. Across the six families, five siblings were available for testing and in all cases variants co-segregated consistent with them being causative. In four families, abnormal alkaline phosphatase results were observed in the direction expected. FACS analysis of knockout HEK293 cells that had been transfected with wild-type or mutant cDNA constructs demonstrated that the variants in *PIGN*, *PIGT* and *PIGO* all led to reduced activity. Splicing assays, performed using leucocyte RNA, showed that a c.336-2A>G variant in *PIGL* resulted in exon skipping and p.D113fs*2. Our results strengthen recently reported disease associations, suggest that defective GPI-anchor biogenesis may explain ~0.15% of individuals with developmental disorders and highlight the benefits of data sharing.

## Introduction

In mammalian cells, there are thought to be over 150 different proteins that are attached to the plasma membrane using a glycosylphosphatidylinositol (GPI) anchor. This diverse family comprises receptors, adhesion molecules and enzymes and is critical for normal neuronal and embryonic development. The GPI anchor is synthesised and remodelled in a complex series of biochemical reactions that take place either in the endoplasmic reticulum (ER) or Golgi apparatus, and at least 30 genes are known that encode components of this pathway.^1, 2^

The clinical significance of this pathway was first demonstrated in 1993 when somatic mutations in *PIGA* (which encodes subunit A of phosphatidylinositol *N*-acetylglucosaminyltransferase) were shown to cause paroxysmal nocturnal haemoglobinuria.^3^ This rare life-threatening disease results from complement-mediated haemolysis due to a deficiency of surface expression of GPI-anchored complement inhibitors CD55 and CD59. At the time, it was speculated that constitutive mutations in this gene would be embryonically lethal, however, this turned out not to be the case and several overlapping phenotypes have now been associated with germline variants.^4, 5, 6, 7, 8^

In 2014, using a combination of exome and targeted gene sequencing, we identified three families where individuals with learning disability and hyperphosphatasia harboured biallelic mutations in *PGAP3.*^9^ Our work, together with results from many other research groups worldwide, have suggested disease associations for at least 18 genes that relate to GPI-anchor biosynthesis (Supplementary Table S1) and the importance of testing this pathway in clinical diagnostics is now increasingly recognised.^2^

Although the phenotype associated with GPI-defects is variable, global developmental delay is the most consistent finding (Supplementary Table S1).^10^ Therefore, seeking to replicate our earlier findings, determine the true incidence of GPI defects in a large unbiased cohort and potentially to identify novel disease–gene associations, we analysed data from the Deciphering Developmental Disorders (DDD) study. This project is a collaboration between the Wellcome Trust Sanger Institute and all 24 Regional Genetics Services in the UK and the Republic of Ireland that aims to facilitate the translation of genomic sequencing technologies into the National Health Service. DDD's analysis of an initial set of 1133 children with severe undiagnosed developmental disorders revealed a genetic variant that is likely to be causative in 317 cases,^11^ which provides considerable scope for providing diagnoses or identifying novel disease genes in the remaining cases. The study has now identified at least 16 new genes responsible for developmental disorders.^12, 13^ Although recruitment to this study ceased in April 2015, with more than 14 000 patients enrolled, the DDD study represents one of the largest exome sequencing initiatives in the world.

## Materials and methods

### Recruitment and patient details

Patient recruitment was undertaken by all Regional Genetics Services in the UK and the Republic of Ireland. Clinical details for the families of interest are summarised in Table 1 and Supplementary Table S2. The DDD study has been described in more detail elsewhere.^11, 12, 13^ More information about the aims of the project, subject recruitment and a list of publications are available at www.ddduk.org.

### Exome analysis and DDD data filtering

Exome sequencing and bioinformatic methods are described in the Supplementary Methods. Potential candidate variants were identified in individuals using VCF files generated by the DDD study and filtering QC-passed variants as follows:

- In an initial data set of 1133 trios, the minor allele frequency (MAF) threshold was 1% for all inheritance models. To improve specificity in the expanded data set of 4293 trios, the MAF threshold for monoallelic variants was reduced to 0.1%.
- Variant effect predictor annotation had to suggest the most severe consequence of the variant is protein altering.
- Inherited missense alterations predicted benign by PolyPhen-2 were excluded.
- Genotype and inheritance had to be consistent with a monoallelic mode (*de novo* or dominantly inherited from affected parent), biallelic mode (homozygous or compound heterozygous) or X-linked mode (hemizygous).

Resulting candidate variants were then filtered for the 31 genes listed in Supplementary Table S1. For trios of interest, a list of all candidate variants was provided. Additional genetic information available included full v4.1 VCFs, annotation for variants that have already been reported back to clinicians via DECIPHER^14^ and a list of Sanger validated *de novo* mutations called by DeNovoGear.^15^ Selected BAM files were downloaded from the European Genome-Phenome Archive (EGA; www.ebi.ac.uk/ega/datasets/EGAD00001001114). Other information included clinical details, which included a list of Human Phenome Ontology terms, information about family relationships and contact details for the referring clinician. Additional information such as VCF files and phenotypic data are available at www.ebi.ac.uk/ega/studies/EGAS00001000775 and the diagnostic variants have been made publicly available through the DECIPHER database:

https://decipher.sanger.ac.uk/patient/257982#genotype

https://decipher.sanger.ac.uk/patient/259633#genotype

https://decipher.sanger.ac.uk/patient/258094#genotype

https://decipher.sanger.ac.uk/patient/270250#genotype

https://decipher.sanger.ac.uk/patient/270306#genotype

https://decipher.sanger.ac.uk/patient/263039#genotype

https://decipher.sanger.ac.uk/patient/277013#genotype

### Re-analysis with alternative genome analysis pipeline

It is well known that there is a low genotype concordance between different variant calling software.^16^ Therefore, data from three families where BAM files were available in EGA were re-analysed with an analysis pipeline that combined multi-sample variant calling with Platypus^17^ and variant prioritisation using Ingenuity Variant Analysis (www.ingenuity.com/products/variant-analysis), similar to that described previously.^18^ For three families where BAM files were not available in EGA at the time of the analysis, we uploaded the VCF files that had been generated from the DDD pipeline to Ingenuity Variant Analysis. We filtered variants looking for both *de novo* and recessive candidate variants using a variety of settings to help confirm that the GPI pathway variants that came up from the primary analysis were the most likely candidates. Read alignments supporting variants of interest were also viewed using the Integrative Genomics Viewer (www.broadinstitute.org/igv).

### Sanger validation

The genomic loci surrounding each of the putative pathogenic variants were PCR amplified using the primers listed in Supplementary Table S3. PCRs were purified using standard methods and bidirectional Sanger sequencing was performed using BigDye chemistry (Applied Biosystems, Foster City, CA, USA).

### Functional analysis of *PIGN, PIGT* and *PIGO* variants

*PIGN-*knockout HEK293 cells were generated and transfected as described previously,^19^ with human wild-type or p.(L311W) mutant *PIGN* cDNA cloned into pME, a strong SRα promoter-driven expression vector, or pTK, a medium TK promoter-driven expression vector. *PIGN* constructs had an HA epitope tag at the N terminus. After 3 days, restoration of the cell surface expression of CD59 was evaluated by flow cytometry. The strong promoter is useful for detecting complete LoF and severe partial LoF, while the medium promoter is helpful for detecting mild partial LoF because overexpression of mild partial LoF mutant often causes full restoration of CD59.

Levels of expressed wild-type and p.(L311W) mutant HA-tagged *PIGN* in pME-vector transfected cells were analysed by western blotting using an anti-HA antibody (C29F4, Cell Signaling Tec, Danvers, MA, USA). Levels of protein expression were normalised by the luciferase activity for transfection efficiencies and by expression levels of GAPDH for loading controls.

*PIGT* and *PIGO*-knockout HEK293 cells were generated by CRISPR/Cas system and the corresponding *PIGT* and *PIGO* variants were assessed by measuring the restoration of CD59 surface expression. Western blotting was used to analyse the protein levels. These experiments were performed as described for *PIGN*, except PIGT cDNA constructs were FLAG-tagged at the C-terminal and probed with anti-FLAG antibody (M2, Sigma-Aldrich, Saint Louis, MO, USA).

### Autozygosity analysis and calculation of inbreeding coefficients

Allelic ratios from a set of high-quality variants were extracted as described in the Supplementary Methods. These data were loaded into Nexus CN (BioDiscovery, El Segundo, CA, USA) to call cnLOH segments across the whole genome. We estimated the coefficient of inbreeding as the total fraction of the autosomal genome, which appeared to be homozygous by descent.

### RNA analysis of *PIGL* splice variant

Fresh blood was collected into PAXgene Blood RNA Tubes and RNA extractions were performed with the PAXGene Blood RNA kit (Qiagen, Manchester, UK). cDNA was reverse transcribed using the QuantiTect kit (Qiagen) and a mixture of oligo-dT and random primers. Forward primers were designed in exons 1 and 2 while reverse primers were designed in exons 5 and 6 (Supplementary Table S3). RT-PCR products were diluted and run on a High Sensitivity DNA Chip on the 2100 Bioanalyzer (Agilent Technologies, Santa Clara, CA, USA). PCR products were also purified using *exo*I (NEB, Ipswich, MA, USA) and shrimp alkaline phosphatase (USB, Cleveland, OH, USA) and Sanger sequencing was performed as described above.

## Results

### Summary of candidates and exclusion criteria

The DDD filtering pipeline identified 43 patient–parent trios (42 independent families and two siblings) in which rare, potentially functional candidate variants were identified in at least one of the GPI-anchor biogenesis genes. As has been noted previously,^11^ parental-affected status significantly influenced the number of candidate variants identified. Across the entire exome, there were on average 65.8 candidate variants in trios where both parents were affected (mostly variants inherited from one or other parent), 34.1 candidates where just a single parent was affected and just 6.7 candidates (range 2–16) where neither parent was affected.

As of July 2015, four of the 43 index cases had variants in other (ie, non-GPI pathway) genes reported that were already considered to be clinically relevant. For instance, a girl with developmental delay and ASD (DECIPHER ID 258536) harboured a *de novo* p.(Q1093*) mutation in *SYNGAP1* (NM_006772.2).^20^

GPI-anchor biogenesis genes reported to date (Supplementary Table S1) are all associated with recessively inherited conditions. We therefore focussed on variants that fitted a biallelic inheritance (ie, compound heterozygous or homozygous) or X-linked recessive models, excluding families where parents were affected and candidate variants fitted a dominant inheritance model.

Focussing on a recessive model also led us to ignore putative *de novo* missense variants in *PIGM* (c.1199A>G; p.(N400S), NM_145167.2) and *MPPE1* (c.682C>T; p.(R228C), NM_023075.5). The inheritance pattern associated with *PIGM* mutations has been reported to be autosomal recessive.^21^ We also note that both these genes have low pLI scores in ExAC v0.3 and so are unlikely to be sensitive to haploinsufficiency.^22^ After further review of candidates, we also excluded a small set of variants, which were detected at MAF 0.1–1.0% but were each present in a homozygous state in ExAC V0.3 multiple times. This led us to exclude patients with biallellic variants in *PIGW* (c.705C>G;705C>G in individual 275308, c.705C>G;908G>A in 259553, NM_178517.3), *PIGS* (c.553C>T;553C>T in 267380, NM_033198.3) and *GPLD1* (c.308A>G;2442delA in 276507, NM_001503.3).

### Overview of likely causative variants

**Figure 1:**
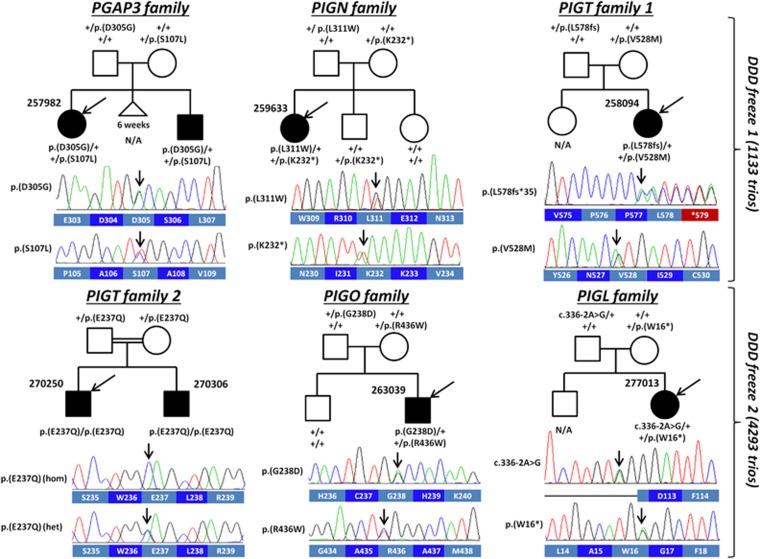
Pedigrees and genetic data for six families harbouring rare biallelic variants in genes encoding components of the GPI-anchor biogenesis pathway. The Sanger sequencing traces shown are for the proband in each family and are shown in the coding direction, alongside the corresponding wild-type amino acid sequence. In the case of *PIGT* family 2 we show a trace from the father, where the variant is in the heterozygous state. For *PIGT* family 1 and the *PIGL* family, DNA was not available for the unaffected older siblings. Codon numbering is with respect to the following GenBank transcripts; *PGAP3*: NM_033419.4; *PIGN*: NM_176787.4; *PIGT*: NM_015937.5; *PIGO*: NM_032634.3; *PIGL*: NM_004278.3.

As a result of the above filtering, potentially clinically significant variants were identified in 7/4293 parent–child trios. These consisted of 11 rare variants spread across five different GPI-anchor biogenesis genes (Figure 1). In five of the families, the variants were in a compound heterozygous configuration. The sixth family was a consanguineous Afghani kindred with two affected brothers and here the likely causative mutation was homozygous.

Including the Afghani quartet, DNA from affected or unaffected siblings was available for testing in four out of six of the families and in all cases, the segregation pattern was consistent with the variants being causative (Figure 1; *P*=0.026). For four out of five genes where alkaline phosphatase testing is known to be informative, abnormal results were obtained and the directionality was as expected, that is, elevated with mutations in three out of five genes, normal with one out of five genes and lowered or close to lower limit with mutations in one out of five genes (Table 1). None of the variants were reported to occur in a homozygous state in ExAC, with total allele counts ranging from 0 to 16 (Table 1).

### *PGAP3* family

Individual 257982 harboured rare compound heterozygous variants in *PGAP3*: a c.914A>G (predicting a p.(D305G) alteration to the amino acid sequence) inherited from the patient's father and a c.320C>T change (predicting p.(S107L)) from the mother. We note that p.(D305G) was described previously (family B in Howard *et al.*^9^) where it was shown to result in abnormal protein localisation to the ER. p.(S107L) was identified in a more recent study where it was shown to reduce PGAP3 activity.^23^ In one case (family D in Knauss *et al.*^23^), the same two variants were identified as in 257982. However, in that patient, p.(S107L) was paternal and p.(D305G) maternal.

Sanger sequencing confirmed that both variants were present in the affected brother of 257982 (Figure 1). In both affected siblings, alkaline phosphatase activity was increased (Table 1), consistent with the results reported previously.^9^

### *PIGN* family

Individual 259633 harboured compound heterozygous variants in *PIGN*: a c.932T>G (predicting p.(L311W)) from the father and a c.694A>T (predicting p.(K232*)) from the mother. Sanger sequencing of two unaffected siblings indicated that neither had inherited both variants (Figure 1). Both variants have been described recently; a homozygous p.(K232*) mutation was seen in a fetus diagnosed with Fryns syndrome,^24^ a condition characterised by multiple congenital anomalies, while p.(L311W) was observed in an individual where the phenotype was limited to hypotonia, developmental delay and seizures.^25^

**Figure 2:**
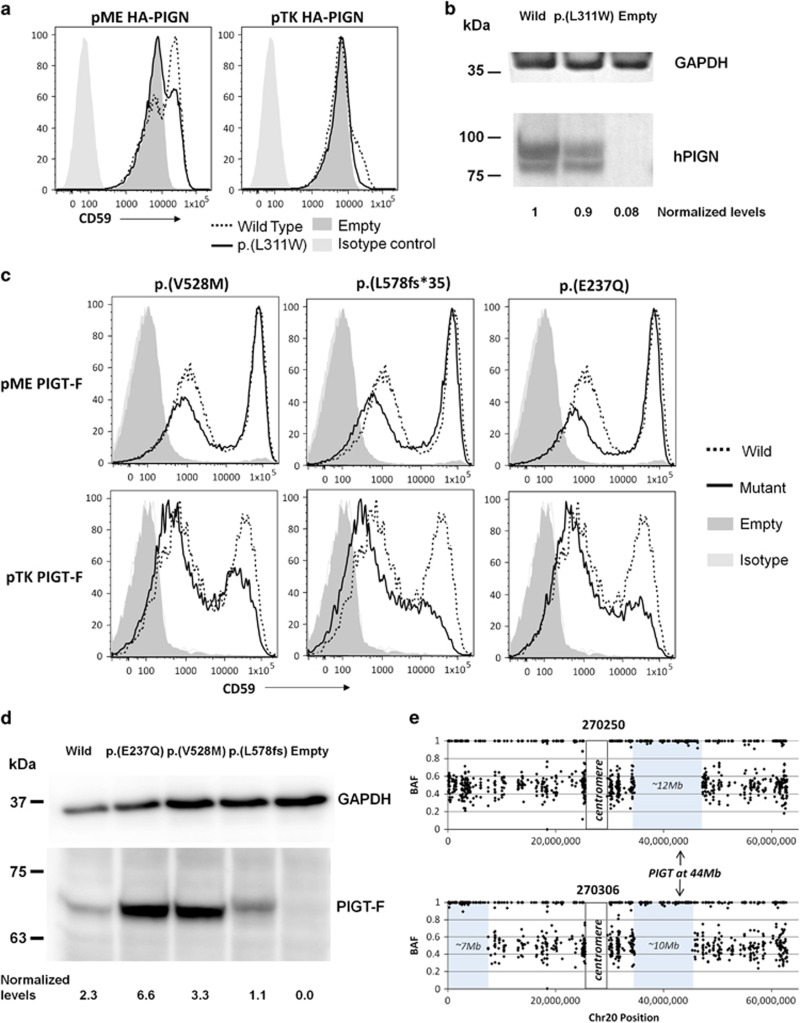
Follow-up studies on variants in *PIGN* and *PIGT*. (**a**) *PIGN-*knockout HEK293 cells were generated and transfected with human wild-type or p.(L311W) mutant *PIGN* cDNA cloned into pME or pTK expression vectors. Restoration of the cell surface expression of CD59 was evaluated by flow cytometry. The mutant construct using the pME promoter did not rescue CD59 surface expression as efficiently as the wild-type construct, indicating that the variant results in reduced PIGN activity. (**b**) Levels of expressed wild-type and p.(L311W) mutant HA-tagged PIGN in pME-vector transfected cells were analysed by western blotting using an anti-HA antibody. After normalisation with luciferase activity and GAPDH, expression of the mutant protein appeared to be reduced by only ~10% compared with the wild-type protein. (**c**) *PIGT-*knockout HEK293 cells were transfected with wild-type or mutant *PIGT* cDNA cloned into pME or pTK expression vectors. Restoration of the cell surface expression of CD59 was evaluated by flow cytometry. The mutant constructs using the pTK promoter did not rescue CD59 surface expression as efficiently as the wild-type construct, indicating that the variants result in reduced PIGT activity. (**d**) Levels of expressed wild-type and mutant FLAG-tagged PIGT in pME-vector transfected cells were analysed by western blotting. After normalisation, expression of the mutant protein appeared to be reduced only for the p.(L578fs*35) variant. (**e**) Allelic ratio plots along chromosome 20 (for high confidence SNVs only) showed that the *PIGT* variant shared in 270250 and 270306 lies within a large region of autozygosity.

Alkaline phosphatase testing for this case is uninformative as normal results are expected for patients with *PIGN* mutations^19, 26^ and therefore functional assessment was performed using PIGN-knockout HEK293 cells. With an expression plasmid using a strong pME promoter, a wild-type PIGN restored CD59 expression on 52% of PIGN-knockout cells after transient transfection, whereas p.(L311W) PIGN restored CD59 on only 39% of the cells (Figure 2a, left panel). With a medium promoter plasmid pTK, the wild-type PIGN restored CD59 on a small fraction of the cells, whereas the p.(L311W) PIGN had almost no effect (right panel). Western blot analysis indicated that the missense alteration did not significantly affect protein expression (Figure 2b). These results indicate that the p.(L311W) mutation reduces enzymatic activity rather than affecting protein levels.

### *PIGT* family 1

Individual 258094 harboured compound heterozygous variants in *PIGT*: c.1582G>A (predicting p.(V528M)) from the mother and c.1730dupC (predicting p.(L578fs*35)) from the father. Sanger sequencing was used to validate both variants, although DNA from the unaffected sister was unavailable for testing. Initial publications on this gene reported decreased alkaline phosphatase activity^27, 28^ but a subsequent study found normal levels.^29^ In this case, alkaline phosphatase activity was in the normal range (Table 1). Rescue experiments performed on PIGT-knockout HEK293 cells indicated that both mutations result in a mild reduction in the amount of CD59 anchored to the cell membrane, although this effect was only seen when using the pTK promoter (Figure 2c). Western blot analysis suggested that p.(L578fs*35) may lead to a small decrease in protein stability (Figure 2d). The functional effect of these two mutations was further confirmed by the reduced CD16 expression seen on patient granulocytes (Supplementary Figure S1).

Recent studies have shown complex multisystem conditions can be a result from blending of two distinct genetic disorders.^30, 31, 32, 33^ In that respect, we note that 258094 also harboured compound heterozygous variants in *PKHD1* (predicting p.(P2319Q); p.(D3923fs*8), NM_138694.3). This gene is associated with Autosomal Recessive Polycystic Kidney Disease (AR-PKD), a severe condition in which a significant fraction of babies die within the first 4 weeks of life due to breathing difficulties. Although 258094 had kidney stones, nephrolithiasis is not typically a feature of AR-PKD.

### *PIGT* family 2

Individual 270250 harboured a homozygous c.709G>C variant (predicting p.(E237Q)) in *PIGT*. An affected brother (270306) was confirmed by both Sanger sequencing and exome analysis to be homozygous for the same variant (Figure 1). Alkaline phosphatase activity for 270250 was below the normal range, whereas for the younger brother it was at the lower end of the normal range (Table 1). FACS analysis of PIGT-knockout HEK293 cells showed that p.(E237Q) results in a small reduction in the amount of CD59 anchored to the cell membrane (Figure 2c).

Using allelic ratio information obtained from the exome data, we estimated the coefficients of inbreeding for 270250 and 270306 to be 1/15 and 1/19 respectively, consistent with the 1/16 theoretical expectation for offspring of first-cousin marriages. The *PIGT* gene was shown to lie within a 10–12 Mb region of autozygosity (Figure 2e). The only larger region of autozygosity shared by both siblings was a 35.5 Mb segment on the short arm of chromosome 2 (data not shown).

### *PIGO* family

**Figure 3:**
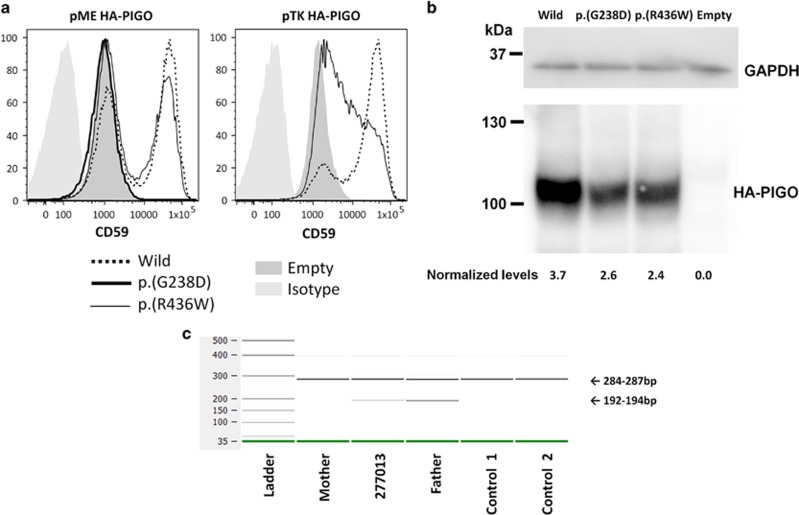
Follow-up studies on variants in *PIGO* and *PIGL.* (**a**) *PIGO-*knockout HEK293 cells were transfected with wild-type, p.(R436W) or p.(G238D) *PIGO* cDNA. Restoration of the cell surface expression of CD59 was evaluated by flow cytometry. The p.(G238D) variant resulted in no detectable activity when using the pME promoter. For the p.(R436W) variant, reduced CD59 surface expression was only observed when using the pTK promoter. (**b**) Levels of expressed wild-type and mutant HA-tagged PIGO in pME-vector transfected cells were analysed by western blotting. After normalisation, expression of the mutant protein appeared to be mildly reduced for both missense variants. (**c**) 2100 Bioanalyser image showing *PIGL* RT-PCR amplicons using primers positioned in exons 2 and 5. A lower band was observed for 277013 and her father, consistent with skipping of exon 3. The expected sizes were calculated to be 280 bp and 189 bp if exon 3 is missing, which is consistent with the observed sizes given the margin for error reported by the manufacturer. Skipping of a 91 bp exon would lead to a frameshift and premature termination codon, as shown in Supplementary Figure S3.

Individual 263039 harboured compound heterozygous variants in *PIGO:* c.1306C>T (predicting p.(R436W)) from the mother and c.713G>A (predicting p.(G238D)) from the father. The unaffected elder brother did not have either variant. Alkaline phosphatase activity was intermittently raised, as is expected.^34^ FACS analysis of PIGO-knockout HEK293 cells showed that p.(G238D) resulted in no detectable activity, consistent with its position within the Type 1 phosphodiesterase/nucleotide pyrophosphatase/phosphate transferase domain and the conservation of Gly238 in known paralogues (PIGN and PIGG). In contrast, p.(R436W) only resulted in a moderate decrease in the amount of CD59 anchored to the cell membrane (Figure 3a). The difference in functional effects could not be explained by protein stability as both missense variants resulted in only a mild decrease in protein expression (Figure 3b).

In addition, an X-linked variant of uncertain significance (c.2683T>A, predicting p.(F895I)) was identified in *BCORL1* (NM_021946.4), a transcriptional co-repressor gene. Although this variant is very rare and not present in ExAC, the evidence supporting *BCORL1* to be a causative gene for learning disability was limited;^35^ many of the proposed genes for X-linked learning disability have recently been challenged in light of data from large exome sequencing data sets.^36^

### *PIGL* family

Individual 277013 harboured compound heterozygous variants in *PIGL:* c.48G>A (predicting p.(W16*)) from the mother and a c.336-2A>G mutation in the exon 3 consensus splice-acceptor site, from the father. DNA from the unaffected brother was unavailable. Alkaline phosphatase results were not reported in the original clinical description of CHIME syndrome^37^ but in a subsequent case with *PIGL* mutations were described to be elevated.^38^ For 277013, alkaline phosphatase levels were persistently raised (Table 1).

RNA analysis of the splice mutation was complicated by the fact that in all the samples, we observed skipping of exon 5, consistent with the Ensembl annotation ENST00000395844. Although this naturally occurring isoform is predicted to result in a LoF allele (p.A166fs*80), we note that this shorter transcript was observed at relatively low levels when compared with the canonical mRNA (Supplementary Figure S2). In view of this, we did not attempt to analyse the exon 3 splice-acceptor mutation using sequence from the ‘6 R' RT-PCR primer. The analysis of RT-PCR products using the ‘5 R' primer demonstrated that the c.336-2A>G mutation resulted in a lower band in both 277013 and in her father (Figure 3c). Sanger sequencing confirmed that this was due to complete skipping of exon 3, predicting a frameshift that results in an aspartic acid to tryptophan alteration followed immediately by a premature stop (p.D113fs*2; Supplementary Figure S3) and therefore likely represents a LoF allele.

Although the stop and splice variants are both seen in ExAC (1/121 332 and 6/121 410, respectively), neither occur in a homozygous state. There were also no other homozygous LoF variants in *PIGL* within ExAC or another project that searched for rare gene knockouts in a cohort enriched for homozygous alleles.^39^

### Overall clinical comparison

**Figure 4:**
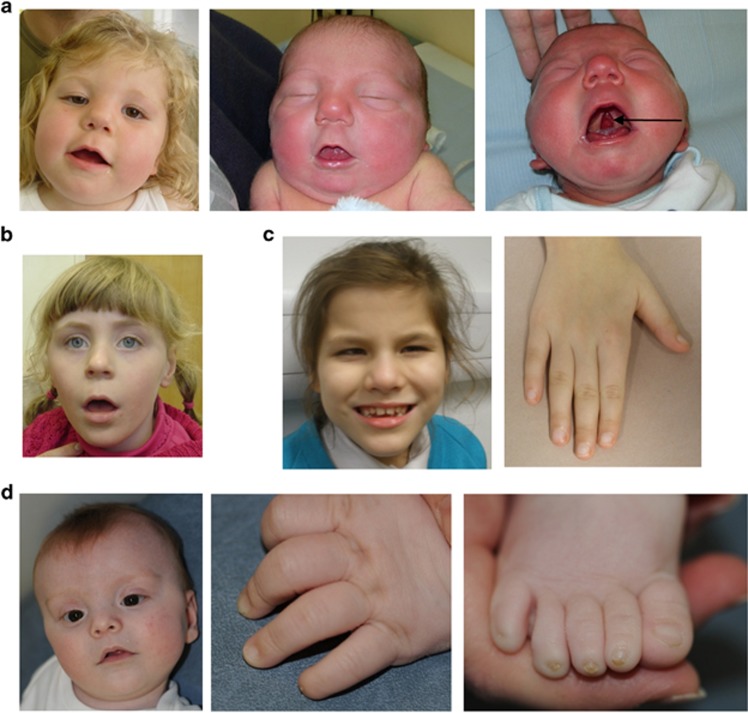
Clinical images, shown with parental consent. (**a**) Photographs of individual 257982 aged 2 years and 8 months and her younger affected brother both showing thin upper lip and short nose with a broad nasal tip. Arrow indicates cleft palate, shown for younger sibling but also present in proband. (**b**) Photograph of 259633 showing thin tented upper lip and a short nose with a broad nasal tip. (**c**) Photographs of 258094 showing thin upper lip, nose with broad nasal tip and low-set ears; hands show tapering fingers. (**d**) Photograph of 263039 showing thin Cupid's-bow shaped upper lip, brachydactyly with absent fifth fingernail and dystrophic fourth and fifth toenails.

Epilepsy and microcephaly was observed in 5/6 and 3/6 of the families, respectively (Table 1). The photographs of patients (Figures 4a–d and data not shown) highlight a number of common facial similarities, most notably the thin tented upper lips and a broad nasal tip apparent in three out of six of the families. Brachydactyly or brachytelephalangy is present in three out of six of the families. This has been previously reported with GPI mutations. Moderate-to-severe intellectual disability is universal. Some patients were noted to have structural brain anomalies such as cerebral atrophy, cerebellar atrophy and Dandy–Walker variant. Other structural abnormalities seen were cleft palate, aganglionic megacolon and renal cysts. Although not individually common, these anomalies have also been previously described.

## Discussion

In this study, we interrogated exome data from 4293 patient–parent trios, looking for rare biallelic variants in 31 genes related to GPI-anchor biogenesis. Seven individuals (from six independent families) were identified, each referred from different Regional Genetics Services across the UK. As the 4293 patients came from 4125 independent families,^40^ we therefore estimate incidence of GPI biogenesis defects in this patient group to be ~0.15% (6/4125). Other studies on GPI-anchor biogenesis have typically either used much tighter patient selection criteria^41^ or else large consanguineous families where genetic mapping is possible.^9^ This is therefore the first study to estimate the prevalence of such defects in a large unbiased cohort with developmental delay.

Together with other recent studies,^23, 42^ our study serves to confirm the genotype–phenotype correlation for *PGAP3* that we first described in 2014.^9^ Besides the elevated alkaline phosphatase, the most noticeable features that overlap the five original cases are the broad nasal tip and thin upper lips, which were seen in both 257982 and her younger brother (Figure 4a). Future studies should test whether the distinct craniofacial gestalt make this a clinically recognisable condition. Midline hand movements similar to those described in family A in Howard *et al*^9^ were reported in the younger brother. Here, the onset of absence and startle seizures was at age 2 years, whereas in published cases, onset was 1.5–23 years and included tonic–clonic and myoclonic forms of epilepsy.^9, 23^ Microcephaly was observed in 3 out of 13 published cases^9^ and in the family described here, a small head size was reported only in the younger brother. Hypotonia was also present in both siblings, consistent with the literature. The p.(D305G) and p.(S107L) mutations have now both been described and so have already been functionally validated.^9, 23^ p.S107L lies close to two other reported mutations (p.(G92D) and p.(P105R)) and so this region of the gene may represent a hotspot for disease-causing mutations.

As well as confirmation of recently reported genotype–phenotype correlations, our study also helps to delineate the phenotypic range associated with certain GPI-anchor biogenesis genes. For instance, Hirschsprung's disease (HD), which is a relatively common feature in cases with ‘hyperphosphatasia with mental retardation syndrome' (HPMRS1) due to *PIGV* mutations (OMIM #239300),^43^ has only been reported in one individual with *PIGO* mutations (HPMRS2; OMIM #614749).^44^ The HD diagnosis for 263039 therefore provides additional evidence that intestinal disorders can be observed across different genetic HPMRS subtypes. Although seizures were not reported (at 2 years of age), in other respects such as the cupid's-bow-shaped upper lip, intermittently elevated alkaline phosphatase, hypoplasia of distal phalanges, postnatal microcephaly and hearing loss, the phenotype for 263039 appears to be similar to published cases.^34, 44, 45^

Mutations in *PIGV* are thought to represent the major cause of ‘hyperphosphatasia with mental retardation syndrome'^43^ and so we were surprised that this gene did not come up in our analysis. We therefore investigated the possibility that we were being overly stringent with our MAF filter. The most common *PIGV* mutation (c.1022C>A; p.(A341E)) is categorised as probably damaging by PolyPhen-2 and present in ~80% of affected families.^43^ However, in ExAC, this variant is seen at a maximum MAF of 17/66 740 alleles (0.025% all heterozygous) within the non-Finnish European population, well below not only the initial 1% cut-off for biallelic variants, but also the 0.1% filter that we applied following manual review of variants.

Although this study has helped replicate relatively new disease genes, all five for which the primary disease association was published since 2011 (Table 1), we were unable to identify likely causative variants in any of the 13 genes in the GPI-anchor biogenesis pathway for which disease associations have not yet been reported. It may be that these genetic conditions are so rare that a larger cohort is needed to identify such families. Alternatively, individuals with variants in other GPI genes might not present with developmental delay. For instance, a recent study suggests that mutations in *PIGC* are embryonically lethal.^46^

One limitation of this study is that missense alterations predicted benign by PolyPhen-2 would be missed. We also excluded variants, which appeared homozygous multiple times within the ExAC cohort. Although we felt these filters were necessary to improve specificity while analysing such a large cohort, it means that our ~0.15% estimate of incidence may represent an underestimate. We also acknowledge that our use of WES (rather than WGS) would miss deep intronic variants or structural variants such as inversions. In particular, we cannot exclude that the *de novo* variants in *PIGM* and *MPPE1* occurred in *trans* with one such variant. Our understanding of GPI-anchor biogenesis in humans may be incomplete. Additional genes involved with this pathway may await discovery and so our candidate gene list should be considered a non-exhaustive list. This could again contribute to an underestimation of the true incidence. Another limitation is that in most cases we were unable to perform FACS analysis to assess levels of GPI-anchored proteins on patient granulocytes, instead relying on phenotypic overlap, segregation testing, alkaline phosphatase activity and functional results from HEK293 cells to accumulate evidence supporting pathogenicity. For all five genes identified, multiple families are already described in the literature. As the phenotypes of the patients described here showed significant overlaps with published cases, we felt that once the variants had been validated, requesting further venepunctures was not warranted. The only exception to this was the girl from PIGT family 1, where alkaline phosphatase results were normal and phenotypic overlap was nonspecific. For this case, FACS analysis of patient granulocytes indicated a mild decrease in surface CD16 levels. For the girl with *PIGN* variants, the clinical overlap with published cases also showed limited specificity. Biallelic variants in *PIGN* cause ‘multiple congenital anomalies-hypotonia-seizures syndrome type 1' (MCAHS1; OMIM 614080).^19, 26^ However, a recent review of published cases highlights significant phenotypic heterogeneity.^24^ Although seizures, developmental delay and hypotonia are always present, other features can include dysmorphisms (low-set ears, micrognathia and distal digital hypoplasia), cerebellar atrophy, nystagmus and diaphragmatic hernia. Therefore, although the phenotype observed for individual 259633 (epilepsy, developmental delay, hypotonia and mild brain atrophy) does overlap, we considered the presentation to be nonspecific. In addition, for *PIGN* mutations, alkaline phosphatase testing is not informative as PIGN deficient individuals do not have hyperphosphatasia. This may be because GPI lacking an EtNP-side branch on Man1 is efficiently added to ALP when GPI transamidase cleaves the GPI-attachment signal sequence.^47^ Using *PIGN-*knockout HEK293 cells, we confirmed that p.(L311W) results in reduced PIGN activity. Jezela-Stanek *et al.*^25^ recently described a similar case with a relatively mild phenotype (seizures, developmental delay and hypotonia) and reduced expression of GPI-APs in patient granulocytes. It is interesting to note that the p.(L311W) variant is also shared in common between these two milder cases. Although p.(L311W) appears to retain some activity, p.(K232*) in contrast is presumably a LoF allele and this might explain why homozygosity of the p.(K232*) variant resulted in the severe prenatal presentation reported recently by McInerney-Leo *et al.*^24^

To facilitate the consistent interpretation of genetic variants between different clinical genetics laboratories, the American College of Medical Genetics and Genomics (ACMG) has developed detailed guidelines about how variants should be interpreted in a systematic way.^48^ Using this scoring system, we classified the 11 variants described in Figure 1 and note that seven of these variants are scored as pathogenic, whereas for four of the variants there is only enough evidence to reach a ‘likely pathogenic' classification (Supplementary Table S4). A recent study showed that even following these recommendations, variant scoring can be inconsistent. Although consensus meetings can improve concordance between laboratories, agreement is not always reached for many variants and further clarifications may be beneficial.^49^ The scoring scheme allows a degree of flexibility and certain criteria can be increased in evidence strength based on expert judgement. For example, both *PGAP3* variants described here have now been described in *trans* with pathogenic variants in three unrelated patients and so the PM3 criteria should be upgraded from moderate to strong. In two cases, we upgraded an inferred classification of ‘likely pathogenic' to ‘pathogenic'. For instance, although the p.(L311W) variant in PIGN has been described before,^25^ this was only in a single affected individual and so we could not invoke PS4, which requires multiple prior observations. But together with the modest co-segregation seen in our family (again, not reaching the level to invoke PP1) and the robust functional experiments performed here using mutant HEK293 cells (Figure 2) and by Jezela-Stanek using patient cells, this was enough to persuade us that this variant is pathogenic.

In conclusion, our study suggests that defective GPI-anchor biogenesis may explain ~0.15% of cases with developmental delay and increases the yield of clinically relevant findings within the DDD patient group that are available for families to help with recurrence risk counselling and potentially the provision of further genetic testing. The results also help confirm and extend the phenotypic range of recently reported disease genes and exemplify the benefits of large scale data sharing, providing a model for other large genomic projects such as the UK's 100 K genomes project.

## Figures and Tables

**Table 1:** Summary of genetic and clinical findings in six families with likely causative variants in genes involved in GPI-anchor biogenesis

	*PGAP3* family	*PIGN* family	*PIGT* family 1	*PIGT* family 2	*PIGO* family	*PIGL* family
Decipher ID	257982	259633	258094	270250	263039	277013
						
Ethnicity and gender (parental relatedness)	Caucasian female (none)	White British Caucasian female (none)	Caucasian female (none)	Afghanistani male (first cousins)	Caucasian male (none)	Caucasian female (none)
						
cDNA; protein annotation (transcript ID)	c.[914A>G][320C>T] p.(D305G);(S107L) (NM_033419.4)	c.[932T>G][694 A>T] p.(L311W);(K232*) (NM_176787.4)	c.[1582G>A][1730dupC] p.(V528M);(L578fs*35) (NM_015937.5)	c.[709G>C][709G>C] p.(E237Q);(E237Q) (NM_015937.5)	c.[1306C>T][713G>A] p.(R436W);(G238D) (NM_032634.3)	c.[48G>A][336-2A>G] p.(W16*);p.D113fs*2 due to skipping of exon 3, see Supplementary Figure S3. (NM_004278.3)
						
Allele frequencies in ExAC V0.3^a^	Not found in ExAC v0.3; 16/96 004	2/38 616; Not found in ExAC v0.3	12/120 996; 3/118 342	8/100 744^b^	1/120 802; Not found in ExAC v0.3	1/121 332; 6/121 410
						
Year disease association published	2014^9^	2011^26^	2013^27^		2012^34^	2012^37^
						
Segregation in siblings (method)	Affected younger brother has both variants (Sanger sequencing).	Neither unaffected siblings are compound heterozygous (Sanger sequencing).	DNA from unaffected older sister not available.	Affected younger brother (270306) has both variants (Sanger and exome sequencing).	Unaffected older brother harbours neither variant (Sanger sequencing).	DNA from unaffected older brother not available.
						
Chances of seeing co-segregation under null hypothesis	1/4	(3/4)^2^	NA	1/4	3/4	NA
						
HPO terms	Bilateral ptosis, widely spaced teeth, wide mouth, pes planus, low-set ears, seizures, generalised neonatal hypotonia, cleft soft palate, Dandy–Walker malformation, moderate-to-severe cognitive impairment	Cognitive impairment, seizures, extrapyramidal dyskinesia	Oculomotor apraxia, absent speech, progressive cerebellar ataxia, ataxia, global developmental delay, motor delay, seizures, nephrolithiasis, cerebellar atrophy	270250: progressive microcephaly, EEG abnormality, seizures, intellectual disability profound, nystagmus, optic atrophy, poor suck 270306: seizures, progressive microcephaly, intellectual disability profound	Aganglionic megacolon, sensorineural hearing impairment, nail dysplasia, brachydactyly, aplastic/hypoplastic fingernail, global developmental delay, microcephaly	Moderate global developmental delay, renal cysts, cutis marmorata, broad hallux, pectus excavatum, wide mouth
						
Alkaline phosphatase result (normal range)	257982: 694 U/l (60–425). Affected brother: 847 U/l (60–425).	199 U/l at 11.5 years, and 208 U/l at 12.5 years (normal range 130–390 U/l) 336 U/l at age 13 years (60–400 U/l).	Have been 119, 120, 119 and 170 U/l (normal range is 70–298 U/l)	270250: Consistently low at 61–93 U/l (rising a little with age). Normal range is 135–530 U/l. 270306: 136 U/l. Normal range is 135–530 U/l.	Intermittently raised: 624 U/l and 418 U/l. Normal range is 60–425 U/l.	Persistently raised: 575 U/l at 1/52 of age 923 U/l at 3/12 819 U/l at 7 years Normal range is 100–400 U/l.
						
Hand or foot abnormalities	257982: Described to have ‘Tapering fingers' Affected brother: Described to have ‘small nails'	No abnormalities reported.	Tapering fingers	NA	Dystrophic 4th and 5th toenails; absent 5th fingernail.	Short fingers, clinodactyly and slightly broad halluces.
						
Microcephaly/OFC and other brain malformations	257982: 55 cm (+0.28 SDs) aged 12 years. MRI at age 2 ½ years detected a mild variant of Dandy–Walker malformation Affected brother: OFC of 51.5 cm aged 6 years (−1.2 SDs). MRI aged one year showed a mild generalised lack of white matter bulk and small olfactory bulbs.	At twelve months OFC on 50th centile At age 6 years, HC on 75th centile. Brain scan indicated mild atrophy.	No microcephaly (53 cm 25–50th, centile aged 9 years). Progressive isolated cerebellar atrophy affecting vermis and cerebellar hemispheres.	270250: Microcephaly 270306: Microcephaly	Reported to be microcephalic aged 2 ½ years.	No microcephaly – OFC 50–75th centile. Brain MRI scan normal at 7 months of age.
						
Seizures	257982: 10 tonic seizures a day aged 2 years. Absence seizures and startle seizures which ceased aged 7/8 years. Affected brother: Absence seizures and startle seizures from the age of 2 years.	Developed epilepsy at age 2 years, which became very severe around age 5, but now (aged 14) is reasonably controlled.	3 febrile convulsions aged 1 year, long fits aged 2 years requiring PICU, generalised tonic–clonic seizures, EEG showed frequent runs of bilateral slow activity intermixed with sharp/spike waves.	270250: Neonatal onset epileptic encephalopathy, with multiple refractory seizures. 270306: As above.	No seizures when last seen aged 2 years.	Brief generalised tonic–clonic seizures from 2 to 6 months of age but none since.

NA, not available; OFC, occipitofrontal circumference. All variants listed have been Sanger validated and are compound heterozygous, except in the case of *PIGT* family 2 for which the variant is homozygous in both affected individuals.

a No homozygous genotypes were observed for any of the variants.

b Eight of 13 986 alleles in South Asian cohort.

## References

[bib1] Kinoshita T: Biosynthesis and deficiencies of glycosylphosphatidylinositol. Proc Jpn Acad Ser B Phys Biol Sci 2014; 90: 130–143. 10.2183/pjab.90.130 PMC4055706 24727937

[bib2] Ng BG, Freeze HH: Human genetic disorders involving glycosylphosphatidylinositol (GPI) anchors and glycosphingolipids (GSL). J Inherit Metab Dis 2015; 38: 171–178. 25164783 10.1007/s10545-014-9752-1 PMC4373530

[bib3] Takeda J, Miyata T, Kawagoe K et al: Deficiency of the GPI anchor caused by a somatic mutation of the PIG-A gene in paroxysmal nocturnal hemoglobinuria. Cell 1993; 73: 703–711. 8500164 10.1016/0092-8674(93)90250-t

[bib4] Belet S, Fieremans N, Yuan X et al: Early frameshift mutation in PIGA identified in a large XLID family without neonatal lethality. Hum Mutat 2014; 35: 350–355. 24357517 10.1002/humu.22498

[bib5] Johnston JJ, Gropman AL, Sapp JC et al: The phenotype of a germline mutation in PIGA: the gene somatically mutated in paroxysmal nocturnal hemoglobinuria. Am J Hum Genet 2012; 90: 295–300. 22305531 10.1016/j.ajhg.2011.11.031 PMC3276655

[bib6] Kato M, Saitsu H, Murakami Y et al: PIGA mutations cause early-onset epileptic encephalopathies and distinctive features. Neurology 2014; 82: 1587–1596. 24706016 10.1212/WNL.0000000000000389

[bib7] Swoboda KJ, Margraf RL, Carey JC et al: A novel germline PIGA mutation in Ferro-Cerebro-Cutaneous syndrome: a neurodegenerative X-linked epileptic encephalopathy with systemic iron-overload. Am J Med Genet A 2014; 164A: 17–28. 24259288 10.1002/ajmg.a.36189 PMC4349522

[bib8] Tarailo-Graovac M, Sinclair G, Stockler-Ipsiroglu S et al: The genotypic and phenotypic spectrum of PIGA deficiency. Orphanet J Rare Dis 2015; 10: 23. 25885527 10.1186/s13023-015-0243-8 PMC4348372

[bib9] Howard MF, Murakami Y, Pagnamenta AT et al: Mutations in PGAP3 impair GPI-anchor maturation, causing a subtype of hyperphosphatasia with mental retardation. Am J Hum Genet 2014; 94: 278–287. 24439110 10.1016/j.ajhg.2013.12.012 PMC3928656

[bib10] Makrythanasis P, Kato M, Zaki MS et al: Pathogenic variants in PIGG cause intellectual disability with seizures and hypotonia. Am J Hum Genet 2016; 98: 615–626. 26996948 10.1016/j.ajhg.2016.02.007 PMC4833197

[bib11] Wright CF, Fitzgerald TW, Jones WD et al: Genetic diagnosis of developmental disorders in the DDD study: a scalable analysis of genome-wide research data. Lancet 2015; 385: 1305–1314. 25529582 10.1016/S0140-6736(14)61705-0 PMC4392068

[bib12] Akawi N, McRae J, Ansari M et al: Discovery of four recessive developmental disorders using probabilistic genotype and phenotype matching among 4125 families. Nat Genet 2015; 47: 1363–1369. 26437029 10.1038/ng.3410 PMC5988033

[bib13] DDD: Large-scale discovery of novel genetic causes of developmental disorders. Nature 2015; 519: 223–228. 25533962 10.1038/nature14135 PMC5955210

[bib14] Firth HV, Richards SM, Bevan AP et al: DECIPHER: Database of Chromosomal Imbalance and Phenotype in Humans Using Ensembl Resources. Am J Hum Genet 2009; 84: 524–533. 19344873 10.1016/j.ajhg.2009.03.010 PMC2667985

[bib15] Ramu A, Noordam MJ, Schwartz RS et al: DeNovoGear: *de novo* indel and point mutation discovery and phasing. Nat Methods 2013; 10: 985–987. 23975140 10.1038/nmeth.2611 PMC4003501

[bib16] Gezsi A, Bolgar B, Marx P, Sarkozy P, Szalai C, Antal P: VariantMetaCaller: automated fusion of variant calling pipelines for quantitative, precision-based filtering. BMC Genomics 2015; 16: 875. 26510841 10.1186/s12864-015-2050-y PMC4625715

[bib17] Rimmer A, Phan H, Mathieson I et al: Integrating mapping-, assembly- and haplotype-based approaches for calling variants in clinical sequencing applications. Nat Genet 2014; 46: 912–918. 25017105 10.1038/ng.3036 PMC4753679

[bib18] Pagnamenta AT, Howard MF, Wisniewski E et al: Germline recessive mutations in PI4KA are associated with perisylvian polymicrogyria, cerebellar hypoplasia and arthrogryposis. Hum Mol Genet 2015; 24: 3732–3741. 25855803 10.1093/hmg/ddv117 PMC4459391

[bib19] Ohba C, Okamoto N, Murakami Y et al: PIGN mutations cause congenital anomalies, developmental delay, hypotonia, epilepsy, and progressive cerebellar atrophy. Neurogenetics 2014; 15: 85–92. 24253414 10.1007/s10048-013-0384-7

[bib20] Parker MJ, Fryer AE, Shears DJ et al: *De novo*, heterozygous, loss-of-function mutations in SYNGAP1 cause a syndromic form of intellectual disability. Am J Med Genet A 2015; 167A: 2231–2237. 26079862 10.1002/ajmg.a.37189 PMC4744742

[bib21] Almeida AM, Murakami Y, Layton DM et al: Hypomorphic promoter mutation in PIGM causes inherited glycosylphosphatidylinositol deficiency. Nat Med 2006; 12: 846–851. 16767100 10.1038/nm1410

[bib22] Lek M, Karczewski KJ, Minikel EV et al: Analysis of protein-coding genetic variation in 60,706 humans. Nature 2016; 536: 285–291. 27535533 10.1038/nature19057 PMC5018207

[bib23] Knaus A, Awaya T, Helbig I et al: Rare non-coding mutations extend the mutational spectrum in the PGAP3 subtype of hyperphosphatasia with mental retardation syndrome. Hum Mutat 2016; 37: 737–744. 27120253 10.1002/humu.23006 PMC5084765

[bib24] McInerney-Leo AM, Harris JE, Gattas M et al: Fryns syndrome associated with recessive mutations in PIGN in two separate families. Hum Mutat 2016; 37: 695–702. 27038415 10.1002/humu.22994

[bib25] Jezela-Stanek A, Ciara E, Piekutowska-Abramczuk D et al: Congenital disorder of glycosylphosphatidylinositol (GPI)-anchor biosynthesis—The phenotype of two patients with novel mutations in the PIGN and PGAP2 genes. Eur J Paediatr Neurol 2016; 20: 462–473. 26879448 10.1016/j.ejpn.2016.01.007

[bib26] Maydan G, Noyman I, Har-Zahav A et al: Multiple congenital anomalies-hypotonia-seizures syndrome is caused by a mutation in PIGN. J Med Genet 2011; 48: 383–389. 21493957 10.1136/jmg.2010.087114

[bib27] Kvarnung M, Nilsson D, Lindstrand A et al: A novel intellectual disability syndrome caused by GPI anchor deficiency due to homozygous mutations in PIGT. J Med Genet 2013; 50: 521–528. 23636107 10.1136/jmedgenet-2013-101654

[bib28] Nakashima M, Kashii H, Murakami Y et al: Novel compound heterozygous PIGT mutations caused multiple congenital anomalies-hypotonia-seizures syndrome 3. Neurogenetics 2014; 15: 193–200. 24906948 10.1007/s10048-014-0408-y

[bib29] Lam C, Golas GA, Davids M et al: Expanding the clinical and molecular characteristics of PIGT-CDG, a disorder of glycosylphosphatidylinositol anchors. Mol Genet Metab 2015; 115: 128–140. 25943031 10.1016/j.ymgme.2015.04.007 PMC6341466

[bib30] Li Y, Salfelder A, Schwab KO et al: Against all odds: blended phenotypes of three single-gene defects. Eur J Hum Genet 2016; 24: 1274–1279. 26813946 10.1038/ejhg.2015.285 PMC4989199

[bib31] Tarailo-Graovac M, Shyr C, Ross CJ et al: Exome sequencing and the management of neurometabolic disorders. N Engl J Med 2016; 374: 2246–2255. 27276562 10.1056/NEJMoa1515792 PMC4983272

[bib32] Yang Y, Muzny DM, Reid JG et al: Clinical whole-exome sequencing for the diagnosis of mendelian disorders. N Engl J Med 2013; 369: 1502–1511. 24088041 10.1056/NEJMoa1306555 PMC4211433

[bib33] Yang Y, Muzny DM, Xia F et al: Molecular findings among patients referred for clinical whole-exome sequencing. JAMA 2014; 312: 1870–1879. 25326635 10.1001/jama.2014.14601 PMC4326249

[bib34] Krawitz PM, Murakami Y, Hecht J et al: Mutations in PIGO, a member of the GPI-anchor-synthesis pathway, cause hyperphosphatasia with mental retardation. Am J Hum Genet 2012; 91: 146–151. 22683086 10.1016/j.ajhg.2012.05.004 PMC3397269

[bib35] Schuurs-Hoeijmakers JH, Vulto-van Silfhout AT, Vissers LE et al: Identification of pathogenic gene variants in small families with intellectually disabled siblings by exome sequencing. J Med Genet 2013; 50: 802–811. 24123876 10.1136/jmedgenet-2013-101644

[bib36] Piton A, Redin C, Mandel JL: XLID-causing mutations and associated genes challenged in light of data from large-scale human exome sequencing. Am J Hum Genet 2013; 93: 368–383. 23871722 10.1016/j.ajhg.2013.06.013 PMC3738825

[bib37] Ng BG, Hackmann K, Jones MA et al: Mutations in the glycosylphosphatidylinositol gene PIGL cause CHIME syndrome. Am J Hum Genet 2012; 90: 685–688. 22444671 10.1016/j.ajhg.2012.02.010 PMC3322218

[bib38] Fujiwara I, Murakami Y, Niihori T et al: Mutations in PIGL in a patient with Mabry syndrome. Am J Med Genet A 2015; 167 A: 777–785. 10.1002/ajmg.a.36987 25706356

[bib39] Narasimhan VM, Hunt KA, Mason D et al: Health and population effects of rare gene knockouts in adult humans with related parents. Science 2016; 352: 474–477. 26940866 10.1126/science.aac8624 PMC4985238

[bib40] McRae JF, Clayton S, Fitzgerald TW et al: Prevalence and architecture of *de novo* mutations in developmental disorders. Nature 2017; 542: 433–438. 28135719 10.1038/nature21062 PMC6016744

[bib41] Krawitz PM, Murakami Y, Riess A et al: PGAP2 mutations, affecting the GPI-anchor-synthesis pathway, cause hyperphosphatasia with mental retardation syndrome. Am J Hum Genet 2013; 92: 584–589. 23561847 10.1016/j.ajhg.2013.03.011 PMC3617374

[bib42] Yavarna T, Al-Dewik N, Al-Mureikhi M et al: High diagnostic yield of clinical exome sequencing in Middle Eastern patients with Mendelian disorders. Hum Genet 2015; 134: 967–980. 26077850 10.1007/s00439-015-1575-0

[bib43] Horn D, Wieczorek D, Metcalfe K et al: Delineation of PIGV mutation spectrum and associated phenotypes in hyperphosphatasia with mental retardation syndrome. Eur J Hum Genet 2014; 22: 762–767. 24129430 10.1038/ejhg.2013.241 PMC4023216

[bib44] Kuki I, Takahashi Y, Okazaki S et al: Vitamin B6-responsive epilepsy due to inherited GPI deficiency. Neurology 2013; 81: 1467–1469. 24049131 10.1212/WNL.0b013e3182a8411a

[bib45] Nakamura K, Osaka H, Murakami Y et al: PIGO mutations in intractable epilepsy and severe developmental delay with mild elevation of alkaline phosphatase levels. Epilepsia 2014; 55: e13–e17. 24417746 10.1111/epi.12508

[bib46] Shamseldin HE, Tulbah M, Kurdi W et al: Identification of embryonic lethal genes in humans by autozygosity mapping and exome sequencing in consanguineous families. Genome Biol 2015; 16: 116. 26036949 10.1186/s13059-015-0681-6 PMC4491988

[bib47] Hong Y, Maeda Y, Watanabe R et al: Pig-n, a mammalian homologue of yeast Mcd4p, is involved in transferring phosphoethanolamine to the first mannose of the glycosylphosphatidylinositol. J Biol Chem 1999; 274: 35099–35106. 10574991 10.1074/jbc.274.49.35099

[bib48] Richards S, Aziz N, Bale S et al: Standards and guidelines for the interpretation of sequence variants: a joint consensus recommendation of the American College of Medical Genetics and Genomics and the Association for Molecular Pathology. Genet Med 2015; 17: 405–424. 25741868 10.1038/gim.2015.30 PMC4544753

[bib49] Amendola LM, Jarvik GP, Leo MC et al: Performance of ACMG-AMP variant-interpretation guidelines among nine laboratories in the Clinical Sequencing Exploratory Research Consortium. Am J Hum Genet 2016; 98: 1067–1076. 27181684 10.1016/j.ajhg.2016.03.024 PMC4908185

