# Supplementary material for: Analysis of exome data for 4293 trios suggests GPI-anchor biogenesis defects are a rare cause of developmental disorders
Source: Eur J Hum Genet. 2017 Mar 22;25(6):669–79. doi: 10.1038/ejhg.2017.32 (PMC5477361; doi:10.1038/ejhg.2017.32)
Supplement: Supplementary Information [file ejhg201732x1.docx]

**Supplementary information**

**Analysis of exome data for 4293 trios suggests GPI-anchor biogenesis defects are a rare cause of developmental disorders**

**Supplementary methods**

**Exome sequencing and bioinformatic analysis**

Methods used in the DDD study have been described in more detail elsewhere.^1-3^ Briefly, DNA samples were extracted from samples collected using the Oragene DNA collection kits, (DNA Genotek, Kanata, ON, Canada) and blood-derived DNA for the proband was provided from the regional genetics laboratories. Exome libraries were prepared using SureSelect RNA baits (Agilent) and sequencing was performed using HiSeq technology (Illumina). Reads were mapped to the reference genome (GRCh37; hs37d5) and PCR duplicates were marked using Picard. Variants were called within the bait regions +/- 100bp using GATK (using the UnifiedGenotyper module), SAMtools and Dindel.

**Extracting high-quality variants for autozygosity analysis**

Allelic ratios from a set of high-quality variants was extracted from the VCF files: these variants were i) non-filtered, ii) at positions sequenced with at least 15x coverage and iii) annotated as known variants (i.e. with an rsID). Multi-allelic sites and indels were also excluded as these classes of variant typically do not give accurate allelic ratio information. The allelic ratio was calculated as the allelic depth for the alternate allele divided by the total depth at that locus. Allelic ratio information was loaded into Nexus CN (BioDiscovery) to call cnLOH segments across the whole genome. We estimated the coefficient of inbreeding as the total fraction of the autosomal genome which appeared to be homozygous by descent.

**Table S1** Genes implicated in GPI anchor biogenesis or degradation, with known clinical associations and genomic locations. NA; no clinical associations reported to our knowledge.

| Gene symbol | Gene name | Clinical associations and primary references | Chromosome |
| --- | --- | --- | --- |
| *DPM1* | Dolichyl-phosphate mannosyltransferase polypeptide 1, catalytic subunit | Severe developmental delay, seizures and dysmorphic features (Congenital Disorder of Glycosylation type 1e)^4,5^ | 20q13.1 |
| *DPM2* | [Dolichyl-phosphate mannosyltransferase polypeptide 2, regulatory subunit](http://www.genenames.org/cgi-bin/gene_symbol_report?hgnc_id=HGNC:3006) | Muscular dystrophy-dystroglycanopathy syndrome with severe epilepsy^6^ | 9q34.13 |
| *DPM3* | Dolichyl-phosphate mannosyltransferase polypeptide 3 | Alpha-dystroglycanopathy^7^ | 1q22 |
| *GPAA1* | Glycosylphosphatidylinositol anchor attachment 1 | NA | 8q24.3 |
| *GPLD1* | Glycosylphosphatidylinositol specific phospholipase D1 | NA | 6p22.1 |
| *MPDU1* | Mannose-P-dolichol utilization defect 1 | Severe psychomotor retardation, seizures, failure to thrive, dry skin and scaling with erythroderma, and impaired vision (Congenital Disorder of Glycosylation type 1f)^8,9^ | 17p13.1-p12 |
| *MPPE1 (PGAP5)* | Metallophosphoesterase 1 | NA | 18p11.21 |
| *PGAP1* | Post-GPI attachment to proteins 1 | Intellectual disability and encephalopathy^10^ | 2q33.1 |
| *PGAP2* | Post-GPI attachment to proteins 2 | Hyperphosphatasia mental retardation syndrome^11,12^ | 11p15.4 |
| *PGAP3* | Post-GPI attachment to proteins 3 | Hyperphosphatasia mental retardation syndrome^13^ | 17q21.2 |
| *PIGA* | Phosphatidylinositol glycan anchor biosynthesis, class A | Somatic mutations can cause paroxysmal nocturnal haemoglobinuria^14^;  Germline mutations can cause cleft palate, neonatal seizures, contractures and central nervous system malformations^15^ | Xp22.1 |
| *PIGB* | Phosphatidylinositol glycan anchor biosynthesis, class B | NA | 15q21.3 |
| *PIGC* | Phosphatidylinositol glycan anchor biosynthesis, class C | Embryonic lethal^16^ | 1q23-q25 |
| *PIGF* | Phosphatidylinositol glycan anchor biosynthesis, class F | NA | 2p21-p16 |
| *PIGG* | Phosphatidylinositol glycan anchor biosynthesis, class G | Intellectual disability, hypotonia, early-onset seizures^17^ | 4p16.3 |
| *PIGH* | Phosphatidylinositol glycan anchor biosynthesis, class H | NA | 14q24.1 |
| *PIGK* | Phosphatidylinositol glycan anchor biosynthesis, class K | NA | 1p31.1 |
| *PIGL* | Phosphatidylinositol glycan anchor biosynthesis, class L | Colobomas, heart defects, ichthyosiform dermatosis, mental retardation and ear anomalies (CHIME syndrome)^18^ | 17p11.2 |
| *PIGM* | Phosphatidylinositol glycan anchor biosynthesis, class M | Venous thrombosis and seizures^19^ | 1q23.2 |
| *PIGN* | Phosphatidylinositol glycan anchor biosynthesis, class N | Multiple congenital anomalies-hypotonia-seizures syndrome^20^ | 18q21.33 |
| *PIGO* | Phosphatidylinositol glycan anchor biosynthesis, class O | Hyperphosphatasia mental retardation syndrome^21^ | 9p13.2 |
| *PIGP* | Phosphatidylinositol glycan anchor biosynthesis, class P | NA | 21q22.2 |
| *PIGQ* | Phosphatidylinositol glycan anchor biosynthesis, class Q | Severe early-onset epilepsy^22,23^ | 16p13.3 |
| *PIGS* | Phosphatidylinositol glycan anchor biosynthesis, class S | NA | 17p13.2 |
| *PIGT* | Phosphatidylinositol glycan anchor biosynthesis, class T | Somatic inactivation can cause paroxysmal nocturnal haemoglobinuria^24^; Germline mutations can cause Intellectual disability^25^ | 20q13.12 |
| *PIGU* | Phosphatidylinositol glycan anchor biosynthesis, class U | NA | 20q11.22 |
| *PIGV* | Phosphatidylinositol glycan anchor biosynthesis, class V | Hyperphosphatasia mental retardation syndrome^26^ | 1p36.11 |
| *PIGW* | Phosphatidylinositol glycan anchor biosynthesis, class W | West syndrome and hyperphosphatasia with mental retardation syndrome^27^ | 17q21.1 |
| *PIGX* | Phosphatidylinositol glycan anchor biosynthesis, class X | NA | 3q29 |
| *PIGY* | Phosphatidylinositol glycan anchor biosynthesis, class Y | Variable phenotype ranging from moderate development  delay and microcephaly to a fatal multi-system disease including dysmorphism, seizures,  severe developmental delay and cataracts^28^ | 4q22.1 |
| *PIGZ* | Phosphatidylinositol glycan anchor biosynthesis, class Z | NA | 3q29 |

**Table S2**  Further details of genetic and clinical findings in 6 families with likely causative mutation in genes involved in GPI anchor biogenesis. OFC, Occipitofrontal Circumference. NA, not available. All variants listed have been Sanger validated and are compound heterozygous, except in the case of *PIGT* family 2 for which the variant is homozygous in both affected individuals.

|  | ***PGAP3* family** | ***PIGN* family** | ***PIGT* family 1** | ***PIGT* family 2** | ***PIGO* family** | ***PIGL* family** |
| --- | --- | --- | --- | --- | --- | --- |
| **Decipher ID** | 257982 | 259633 | 258094 | 270250 | 263039 | 277013 |
| **hg19 annotation** | chr17:g.37829105T>C  chr17:g.37840962G>A | chr18:g.59810570A>C  chr18:g.59814315T>A | chr20:g.44054311G>A  chr20:g.44054459dupC | chr20:g.44048803G>C  (homozygous) | chr9:g.35092578G>A  chr9:g.35093964C>T | chr17:g.16120588G>A  chr17:g.16203200A>G |
| **Potential diagnoses proposed** | Myotonic Dystrophy, Ritscher Schinzel,  22q 13 deletion syndrome, Carey Fineman Ziter, Le Marec | Myotonic dystrophy, Spinocerebellar ataxia | Ataxia with oculomotor apraxia syndrome | Described as a probable neurometabolic / neurogenetic disorder | Hirschsprung’s | NA |
| **Pregnancy** | Uneventful pregnancies. Both children born by elective Caesarean section.  Affected brother:  A fetal MRI scan at 21 weeks noted a small cerebellum (normal at 32 weeks) | NA | PV bleeding at 6-8/40 and 12/40 | NA | Normal | Normal  Breech position for delivery |
| **Birth measurements** | 257982:  Born at 38 weeks weighing 3.48kg (50^th^ centile), head circumference on 90^th^ centile  Affected brother:  NA | Born at 39 weeks weighing 4.20kg | Born at 42 weeks weighing 3.99kg (91^st^ centile) | 270250:  Born at 41 weeks weighing 2.68kg, OFC of 35.5cm (59^th^ centile)  270306:  Born at 40 weeks weighing 3.66kg | Born at 40 weeks weighing 3.65kg, with an OFC of 34.3cm (24^th^ centile) | Born at 39 weeks weighing 4.37kg |
| **Dysmorphic features** | Both siblings have plagiocephaly, low set ears, a smooth philtrum, widely spaced teeth, thin upper lip and a broad nasal base.  257982:  Prominent epicanthic folds, hypertelorism, inverted nipples  Affected brother:  a short neck, posteriorly-rotated ears, a prominent occiput. | Facially with strong resemblance to parents. | Slightly deepset eyes and broad nasal tip. | No congenital malformations in either sibling. | Thin cupid’s-bow shaped upper lip. | Widely spaced peg like teeth, wide mouth. |
| **Height, weight** | 257982:  Weight and head circumference on the 50th centile and height 9-25th centile aged 2 years  130cm (-3.56 SDs), 35.7kg (-1.4 SDs) aged 12 years  Affected brother:  Height 107.5cm (-2.3 SDs) and weights 17.4 kg (-1.9 SDs) aged 6 years. | At twelve months, weight on 50^th^ and height on 98^th^ centile.  At age 6, weight on 50^th^ and height on 25^th^ centile. | 135cm (50^th^-75^th^ centile) and 32.4kg (75^th^ centile) at age of 9 years | 270250:  NA  270306:  28kg aged 5 and 1/2 years (99 centile) | NA | Growth parameters all 50^th^-75^th^ |
| **Cleft palate** | 257982:  cleft palate, repaired at 6 months  Affected brother:  Soft palate cleft | No | No | No | NA | No |
| **Development:**  **Speech** | 257982:  Still no real words aged 12  Affected brother:  no words and unable to sign aged 6 ½ years | No speech aged 15 | Developmental delay (limited by ataxia), single words aged 9 years. Has dysarthia and just 20 clear words. Understanding a lot better than speech. At mainstream school with one-to-one support. | Profound neurodevelopmental delay | No speech aged 5 years | General developmental delay but particularly speech. At age 5 was having 13 hours 1 to 1 with a teaching assistant, as well as speech therapy. |
| **Motor skills** | 257982:  Not rolled until 9 months, started walking aged 6 years but at 13 years still very unsteady with a broad based gait and frequently falls over.  Affected brother:  Able to sit with support by six months, walks with support aged 6 ½ years | Limited to cruising around furniture | Sitting unaided at 18 months, walking unaided at 48 months, ataxic gate, mildly increased upper limb tone | NA | Rolls over. Age 5 years he is not able to sit independently or walk. | Sitting at 11 months, walking at 18 months |
| **Other problems** | 257982:  kyphoscoliosis with a lumbar lordosis, very hypermobile bilateral ptosis and recurrent otitis media  Affected brother:  Right-sided ptosis, very hypermobile, bilateral glue ear aged 2 years, involuntary midline  hand movements | Severe chest infections | Renal stones, upward deviation of eyes and poor visual fixation noted at 4 weeks of age, “rolling eyes and trembling chin” noted shortly after birth, oculomotor apraxia | 270250:  Axonal sensorimotor neuropathy, Orobulbar dysfunction – currently on nasogastric feeds – awaiting gastrostomy, Adenotonsillectomy 270306:  As above | Failed newborn hearing screen; vomiting at 2 days old | Cystic upper pole of right kidney, conductive hearing loss treated with grommets, skin dry, cutis marmorata, one strawberry naevus, mixed conductive/sensorineural hearing loss treated with grommets |
| **Previous investigations** | 257982:  Normal karyotype, aCGH (44k resolution) results. FISH ruled out Di George syndrome and normal telomeres. FGFR3, 15q methylation, cholesterol synthesis and protein glycosylation all normal.  Affected brother:  karyotyping, 22q11 deletion, amino acids, thyroid function, ammonia, CK, and toxoplasma and CMV antibodies all normal. | Normal karyotype (blood and skin) and microarray. Normal mutation testing for myotonic dystrophy, Friedrich’s ataxia,UBE3A, DRPLA, SCA1,2,3,6,7, SCN1A, and MECP2 normal. Normal WCE’s, lactate in blood and CSF, transferrin isoelectric focussing, urine amino acid and organic acid and MPs screening. Normal copper and ceruloplasmin. Normal hearing | Normal microarray results (but not karyotyped), normal transferrin glycoforms, normal white cell and plasma enzymes, normal VLCFA, alpha fetoprotein raised on some occasions, ataxia telangiectasia testing normal | NA | Normal array CGH and metabolic tests | Normal array CGH, metabolic investigations and normal FISH result for Pallister-Killian on buccal smear |

**Table S3** Primer sequences used for Sanger validation, segregation testing and RNA analysis. ^+^both variants in *PIGT* family 1 lie in this exon.

| **Primer name** | **Primer sequence** | **PCR size** |
| --- | --- | --- |
| *For validation and segregation testing of genomic DNA* | | |
| *PGAP3-3F* | 5’-GGTAGACAGAGGGCTTCAGG-3’ | 386bp |
| *PGAP3-3R* | 5’-ccaaactccagagctgagga-3’ |  |
| *PGAP3-8F* | 5’-GGTAGACAGAGGGCTTCAGG-3’ | 574bp |
| *PGAP3-8R* | 5’-TTCCTTGCCCACTCCTTAGG-3’ |  |
| *PIGN-L311W-F* | 5’-CACAAGAGAGATTAGCTGGGC-3’ | 305bp |
| *PIGN-L311W-R* | 5’-TCCATAAGTATTGGCCACTCC-3’ |  |
| *PIGN-K232*F* | 5’-ACACACTAACCCCAGTCTGT-3’ | 365bp |
| *PIGN-K232*R* | 5’-TCCCCTCCTTTTAAGCCATCT-3’ |  |
| *PIGT-12F* | 5’-tcctgtctcaccgctcttc-3’ | 359bp^+^ |
| *PIGT-12R* | 5’-caagtggcagaaacagccc-3’ |  |
| *PIGT-6F* | 5’-ACATAGATGAGGGATTGAGTCTG-3’ | 244bp |
| *PIGT-6R* | 5’-CCACATAGATAAGGCAGGGC-3’ |  |
| *PIGO-4F* | 5’-GTGTCAAAGCCTGGCTGG-3’ | 363bp |
| *PIGO-4R* | 5’-ATCCTGATGCCCAAAGCTAC-3’ |  |
| *PIGO-7F_1* | 5’-TCTTAGTCCCACCTTCCATACC-3’ | 637bp |
| *PIGO-7R_1* | 5’-CCACAGAAAAGGGAGGAATG-3’ |  |
| *PIGL-* *1334F* | 5’-ACTGGAGCTTAGGGAGAAGG-3’ | 248bp |
| *PIGL-1334R* | 5’-GGAGGGTGGCCACAGAAAA-3’ |  |
| *PIGL-1335F* | 5’-CAGCCGGAGTCAGGGTCA-3’ | 396bp |
| *PIGL-1335R* | 5’-CCCTACGGAAAACTTCGAGAA-3’ |  |
| *For splicing assay* | | |
| *PIGL-1F* | 5’-GGTGTACCTGCTTTGCTTCT-3’ | 374bp |
| *PIGL-6R* | 5’-GAAGGGAGATGTACTTGCGC-3’ |  |
| *PIGL-2F* | 5’-ACTACAATCAAGGAGAGACTCGT-3’ | 280bp |
| *PIGL-5R* | 5’-GGTAACTTCCCTTCTGAGTGC-3’ |  |

**Figure S1** FACS analysis of CD16 expression in granulocytes from the proband in PIGT family 1. Blood samples from the proband and a female control were treated with ACK lysis buffer and stained with anti-human CD16 (Invitrogen). Samples were run on a BD FACSCanto and data analysed by FlowJo. Granulocytes were identified according to FSc and SSc profile. A mild reduction in the expression of CD16 was observed. FMO, fluorescence minus one.

**
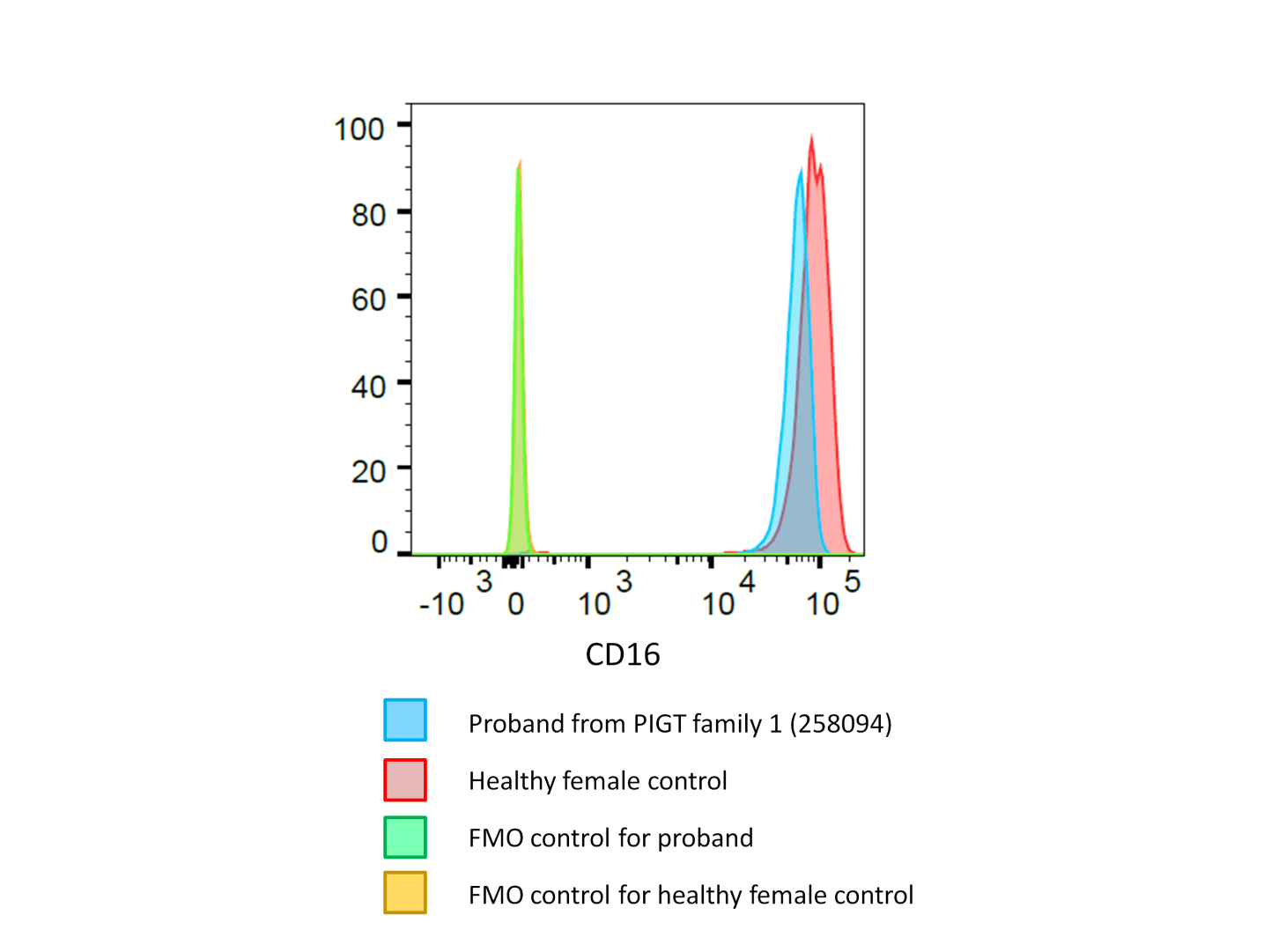
**

**Figure S2** Alternative splicing of *PIGL* exon 5 was detected in controls, consistent with Ensembl transcript annotation ENST00000395844. Therefore confirmation of exon 3 skipping using primer 6R was not attempted. The Sanger trace shown is generated from primer 1F. The position of an alternative primer “5R” used in subsequent experiments is shown.


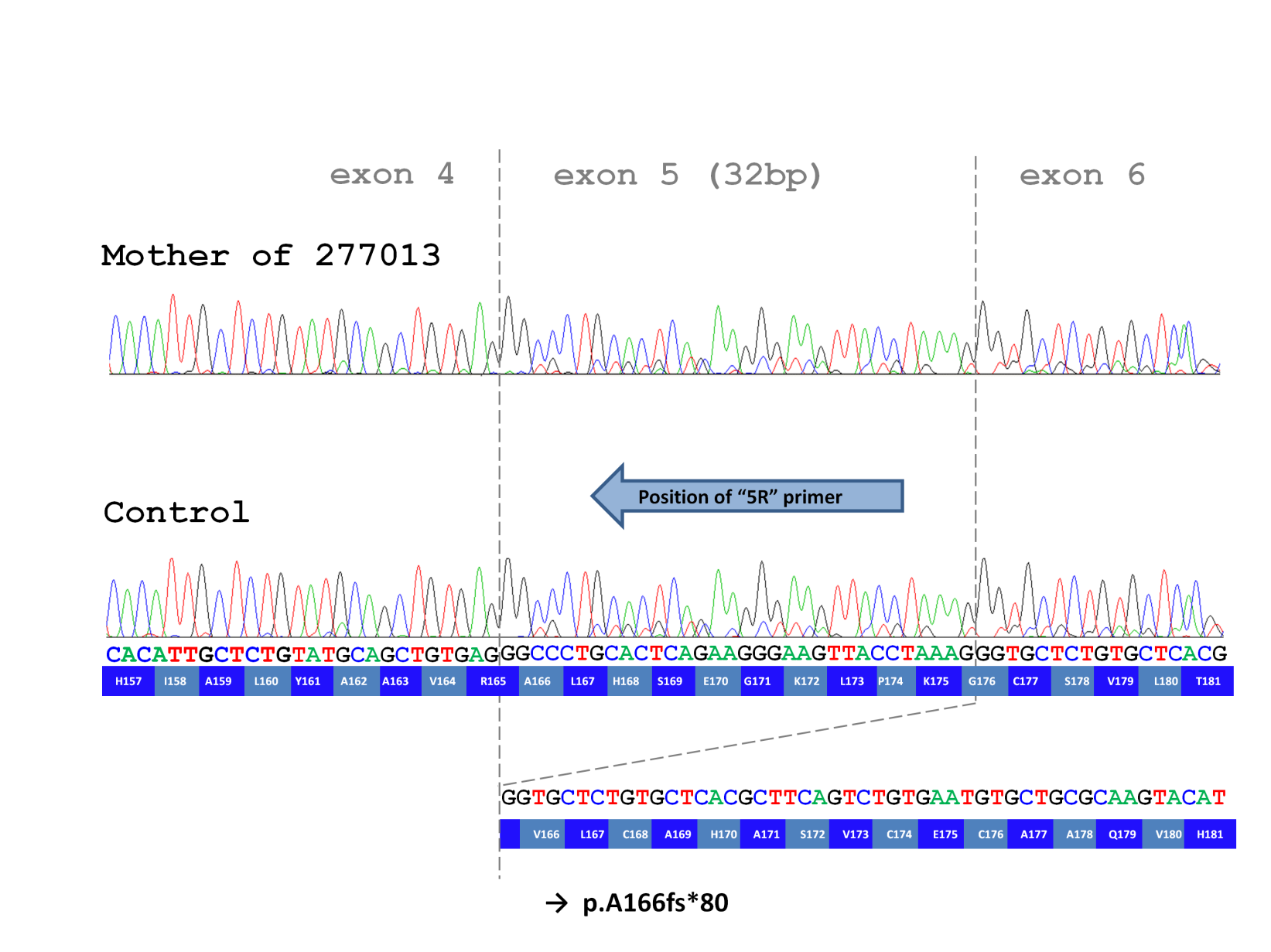


**Figure S3** The *PIGL* splice acceptor mutation (c.336-2A>G) appears to result in skipping of exon 3. Sanger trace shown is generated from primer 1F as 2F was too close to breakpoint to get good sequence. Similar results were obtained using primer 5R.


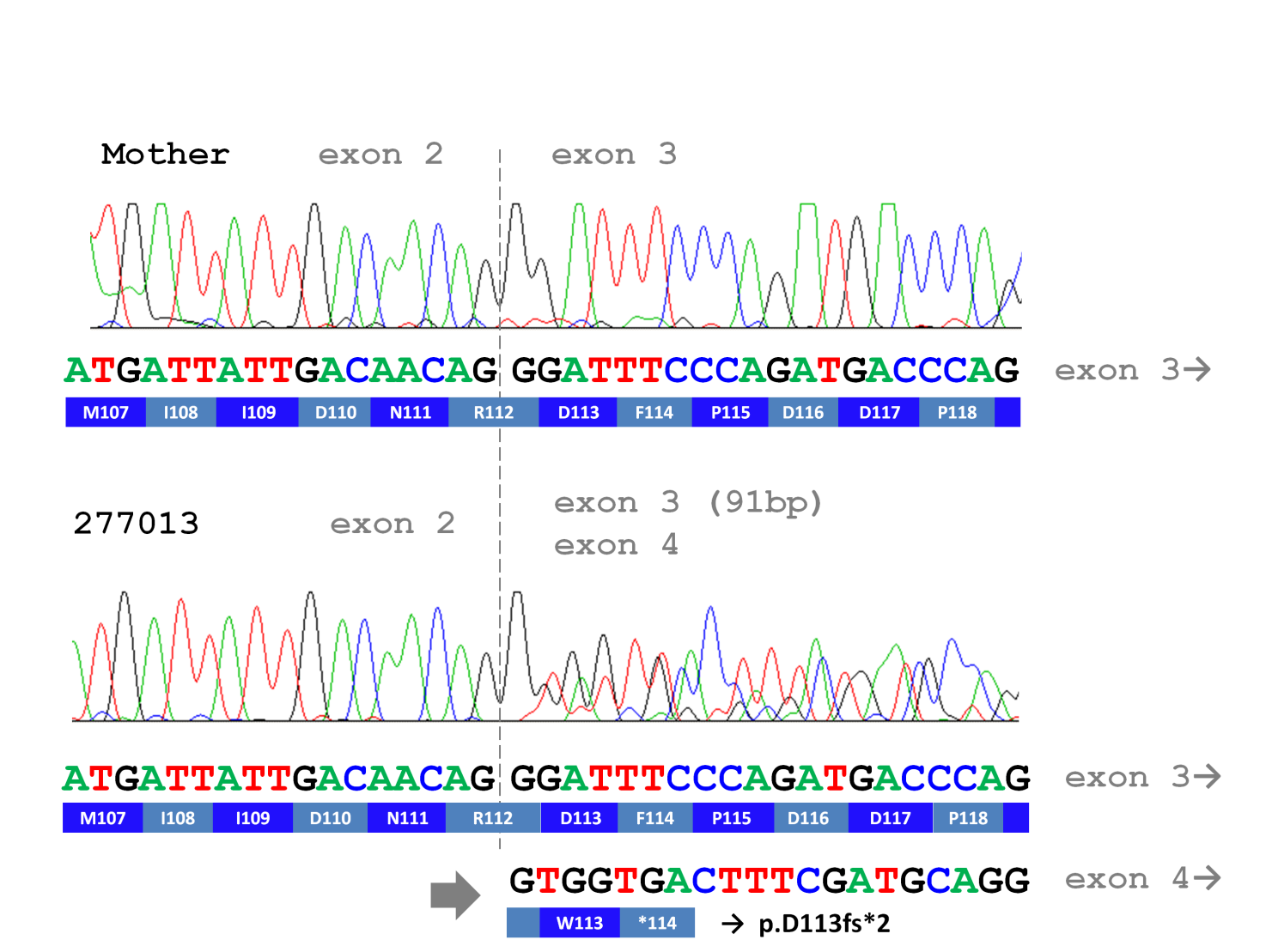


**REFERENCES**

1. Wright CF, Fitzgerald TW, Jones WD, et al. Genetic diagnosis of developmental disorders in the DDD study: a scalable analysis of genome-wide research data. *Lancet* 2015;**385**(9975):1305-14.

2. Akawi N, McRae J, Ansari M, et al. Discovery of four recessive developmental disorders using probabilistic genotype and phenotype matching among 4,125 families. *Nat Genet* 2015;**47**(11):1363-9.

3. DDD. Large-scale discovery of novel genetic causes of developmental disorders. *Nature* 2015;**519**(7542):223-8.

4. Imbach T, Schenk B, Schollen E, et al. Deficiency of dolichol-phosphate-mannose synthase-1 causes congenital disorder of glycosylation type Ie. *J Clin Invest* 2000;**105**(2):233-9.

5. Kim S, Westphal V, Srikrishna G, et al. Dolichol phosphate mannose synthase (DPM1) mutations define congenital disorder of glycosylation Ie (CDG-Ie). *J Clin Invest* 2000;**105**(2):191-8.

6. Barone R, Aiello C, Race V, et al. DPM2-CDG: a muscular dystrophy-dystroglycanopathy syndrome with severe epilepsy. *Ann Neurol* 2012;**72**(4):550-8.

7. Lefeber DJ, Schonberger J, Morava E, et al. Deficiency of Dol-P-Man synthase subunit DPM3 bridges the congenital disorders of glycosylation with the dystroglycanopathies. *Am J Hum Genet* 2009;**85**(1):76-86.

8. Schenk B, Imbach T, Frank CG, et al. MPDU1 mutations underlie a novel human congenital disorder of glycosylation, designated type If. *J Clin Invest* 2001;**108**(11):1687-95.

9. Kranz C, Denecke J, Lehrman MA, et al. A mutation in the human MPDU1 gene causes congenital disorder of glycosylation type If (CDG-If). *J Clin Invest* 2001;**108**(11):1613-9.

10. Murakami Y, Tawamie H, Maeda Y, et al. Null mutation in PGAP1 impairing Gpi-anchor maturation in patients with intellectual disability and encephalopathy. *PLoS Genet* 2014;**10**(5):e1004320.

11. Hansen L, Tawamie H, Murakami Y, et al. Hypomorphic mutations in PGAP2, encoding a GPI-anchor-remodeling protein, cause autosomal-recessive intellectual disability. *Am J Hum Genet* 2013;**92**(4):575-83.

12. Krawitz PM, Murakami Y, Riess A, et al. PGAP2 mutations, affecting the GPI-anchor-synthesis pathway, cause hyperphosphatasia with mental retardation syndrome. *Am J Hum Genet* 2013;**92**(4):584-9.

13. Howard MF, Murakami Y, Pagnamenta AT, et al. Mutations in PGAP3 impair GPI-anchor maturation, causing a subtype of hyperphosphatasia with mental retardation. *Am J Hum Genet* 2014;**94**(2):278-87.

14. Takeda J, Miyata T, Kawagoe K, et al. Deficiency of the GPI anchor caused by a somatic mutation of the PIG-A gene in paroxysmal nocturnal hemoglobinuria. *Cell* 1993;**73**(4):703-11.

15. Johnston JJ, Gropman AL, Sapp JC, et al. The phenotype of a germline mutation in PIGA: the gene somatically mutated in paroxysmal nocturnal hemoglobinuria. *Am J Hum Genet* 2012;**90**(2):295-300.

16. Shamseldin HE, Tulbah M, Kurdi W, et al. Identification of embryonic lethal genes in humans by autozygosity mapping and exome sequencing in consanguineous families. *Genome Biol* 2015;**16**(1):116.

17. Makrythanasis P, Kato M, Zaki MS, et al. Pathogenic Variants in PIGG Cause Intellectual Disability with Seizures and Hypotonia. *Am J Hum Genet* 2016;**98**(4):615-26.

18. Ng BG, Hackmann K, Jones MA, et al. Mutations in the glycosylphosphatidylinositol gene PIGL cause CHIME syndrome. *Am J Hum Genet* 2012;**90**(4):685-8.

19. Almeida AM, Murakami Y, Layton DM, et al. Hypomorphic promoter mutation in PIGM causes inherited glycosylphosphatidylinositol deficiency. *Nat Med* 2006;**12**(7):846-51.

20. Maydan G, Noyman I, Har-Zahav A, et al. Multiple congenital anomalies-hypotonia-seizures syndrome is caused by a mutation in PIGN. *J Med Genet* 2011;**48**(6):383-9.

21. Krawitz PM, Murakami Y, Hecht J, et al. Mutations in PIGO, a member of the GPI-anchor-synthesis pathway, cause hyperphosphatasia with mental retardation. *Am J Hum Genet* 2012;**91**(1):146-51.

22. Alazami AM, Patel N, Shamseldin HE, et al. Accelerating novel candidate gene discovery in neurogenetic disorders via whole-exome sequencing of prescreened multiplex consanguineous families. *Cell Rep* 2015;**10**(2):148-61.

23. Martin HC, Kim GE, Pagnamenta AT, et al. Clinical whole-genome sequencing in severe early-onset epilepsy reveals new genes and improves molecular diagnosis. *Hum Mol Genet* 2014;**23**(12):3200-11.

24. Krawitz PM, Hochsmann B, Murakami Y, et al. A case of paroxysmal nocturnal hemoglobinuria caused by a germline mutation and a somatic mutation in PIGT. *Blood* 2013;**122**(7):1312-5.

25. Kvarnung M, Nilsson D, Lindstrand A, et al. A novel intellectual disability syndrome caused by GPI anchor deficiency due to homozygous mutations in PIGT. *J Med Genet* 2013;**50**(8):521-8.

26. Krawitz PM, Schweiger MR, Rodelsperger C, et al. Identity-by-descent filtering of exome sequence data identifies PIGV mutations in hyperphosphatasia mental retardation syndrome. *Nat Genet* 2010;**42**(10):827-9.

27. Chiyonobu T, Inoue N, Morimoto M, Kinoshita T, Murakami Y. Glycosylphosphatidylinositol (GPI) anchor deficiency caused by mutations in PIGW is associated with West syndrome and hyperphosphatasia with mental retardation syndrome. *J Med Genet* 2014;**51**(3):203-7.

28. Ilkovski B, Pagnamenta AT, O'Grady GL, et al. Mutations in PIGY: expanding the phenotype of inherited glycosylphosphatidylinositol deficiencies. *Hum Mol Genet* 2015;**24**(21):6146-59.
